# Retraction: Resveratrol attenuates inflammation and reduces matrix-metalloprotease expression by inducing autophagy *via* suppressing the Wnt/β-catenin signaling pathway in IL-1β-induced osteoarthritis chondrocytes

**DOI:** 10.1039/d1ra90015c

**Published:** 2021-01-20

**Authors:** Laura Fisher

**Affiliations:** Royal Society of Chemistry Thomas Graham House, Science Park, Milton Road Cambridge CB4 0WF UK advances-rsc@rsc.org

## Abstract

Retraction of ‘Resveratrol attenuates inflammation and reduces matrix-metalloprotease expression by inducing autophagy *via* suppressing the Wnt/β-catenin signaling pathway in IL-1β-induced osteoarthritis chondrocytes’ by Ci Li *et al.*, *RSC Adv.*, 2018, **8**, 20202–20210, DOI: 10.1039/C8RA00993G.

The Royal Society of Chemistry hereby wholly retracts this *RSC Advances* article due to concerns with the reliability of the data.

The images in the article were screened by an image integrity expert who confirmed that some of the western blots images in this paper had been duplicated in another article by a different set of authors.

The control bands (GAPDH) in Fig. 1E of this paper are identical to the western blot control bands (GAPDH) presented in Fig. 3A of ref. 1.

The authors were asked to provide the raw data for this article but did not respond. Given the significance of the concerns about the validity of the data, and the lack of raw data, the findings presented in this paper are not reliable.

The authors have been informed but have not responded to any correspondence regarding the retraction.

Signed: Laura Fisher, Executive Editor, *RSC Advances*

Date: 7^th^ January 2021

## Supplementary Material
